# Impact of Anti-EGFR Therapies on HER2-Positive Metastatic Colorectal Cancer: A Systematic Literature Review and Meta-Analysis of Clinical Outcomes

**DOI:** 10.1093/oncolo/oyad200

**Published:** 2023-07-18

**Authors:** Tanios S Bekaii-Saab, Krzysztof Lach, Ling-I Hsu, Muriel Siadak, Mike Stecher, James Ward, Rachel Beckerman, John H Strickler

**Affiliations:** Division of Hematology/Oncology, Mayo Clinic, Phoenix, AZ, USA; Maple Health Group, LLC, New York, NY, USA; Seagen Inc., Bothell, WA, USA; Seagen Inc., Bothell, WA, USA; Seagen Inc., Bothell, WA, USA; Seagen Inc., Bothell, WA, USA; Maple Health Group, LLC, New York, NY, USA; Division of Medical Oncology, Duke University Medical Center, Durham, NC, USA

**Keywords:** metastatic colorectal cancer, epidermal growth factor receptor, human epidermal growth factor receptor 2, meta-analysis

## Abstract

**Background:**

HER2 overexpression/amplification in patients with RAS wild-type (WT) metastatic colorectal cancer (mCRC) may be associated with resistance to standard-of-care anti-EGFR therapies. Given the lack of comprehensive investigations into this association, we assessed the prognostic or predictive effect of HER2 amplification/overexpression on anti-EGFR treatment outcomes.

**Methods:**

A systematic review of MEDLINE, Embase, and Cochrane Library (2001-2021) identified studies evaluating progression-free survival (PFS), overall response rate (ORR), and overall survival (OS) in HER2-positive vs. HER2-negative patients with RAS WT mCRC who received anti-EGFR treatments and whose HER2 status was known. Meta-analyses of proportions (ORR) and hazard ratios (PFS, OS) were performed using random-effect models with pre-specified sensitivity analyses.

**Results:**

Five high-quality retrospective cohort studies were included in the meta-analyses representing 594 patients with mCRC. All patients received anti-EGFR treatment, either as monotherapy or in combination with chemotherapy. Meta-analysis of PFS demonstrated a 2.84-fold higher risk of death or progression (95% CI, 1.44-5.60) in patients with HER2-positive (vs. HER2-negative) RAS WT mCRC treated with anti-EGFR regimens. The odds of response to anti-EGFR treatment were 2-fold higher in HER2-negative vs. HER2-positive (odds ratio, 1.96 [95% CI, 1.10-3.48]). Differences in OS were not statistically significant. Sensitivity analyses confirmed the robustness of the base-case estimates.

**Conclusions:**

While this study could not account for all confounding factors, in patients with RAS WT mCRC who received anti-EGFR therapy, HER2 overexpression/amplification was associated with worse PFS and ORR and may therefore predict poorer outcomes. HER2 testing is important to inform treatment decisions and could optimize outcomes for patients.

## Teaser

This review assesses current evidence on the prognostic or predictive effect of HER2 amplification/overexpression on anti-EGFR treatment outcomes in patients with RAS wild-type metastatic colorectal cancer.

**Implications for Practice**

In patients with metastatic colorectal cancer (mCRC), overexpression/amplification of HER2 may lead to resistance to treatments targeted to epidermal growth factor receptor (EGFR). We conducted a systematic review and meta-analysis of studies that measured survival and responses in HER2-positive, compared with HER2-negative, patients with mCRC who received anti-EGFR therapy. Patients with HER2-positive mCRC had a 2.84 times higher risk of death or disease progression than patients with HER2-negative mCRC. This finding suggests patients with HER2-positive mCRC may have poorer outcomes with anti-EGFR therapy and highlights the importance of HER2 testing.

## Introduction

Globally, colorectal cancer (CRC) was the third most common cancer and the second leading cause of cancer death in 2020, accounting for 1.9 million new cancer cases and over 935 000 deaths.^1^ Approximately 20%-25% of patients with CRC present with advanced/metastatic stage at the time of diagnosis.^2^ Comprehensive genomic profiling has become crucial in informing treatment decisions and improving outcomes in metastatic colorectal cancer (mCRC), such that the current NCCN Clinical Practice Guidelines in Oncology (NCCN Guidelines) now recommend testing patients for *KRAS/NRAS* and *BRAF*^*V600E*^ mutations, MSI-H/dMMR status, *HER2* amplification, and *NTRK* gene fusions.^3,4^

The activation of the downstream pathways of the epidermal growth factor receptor (EGFR) plays a pivotal role in mCRC development and progression.^5,6^ Cetuximab and panitumumab are anti-EGFR monoclonal antibodies and have been used routinely, either alone or in combination with chemotherapy, in patients with mCRC.^7^ However, pathogenic *RAS* mutations (which include *KRAS/NRAS*) have been shown to drive resistance to anti-EGFR therapies.^8-10^ At the same time, not all patients with *RAS* wild-type (WT) will respond to anti-EGFR regimens.^11,12^

Human epidermal growth factor receptor 2 (HER2) is overexpressed/amplified in 3%-5% of patients with mCRC and in 5%-14% of patients with RAS WT mCRC.^13^ HER2 can activate downstream effectors of EGFR to trigger the signaling pathways while bypassing EGFR.^6^ As such, *HER2* gene amplification allows for the activation of downstream signaling even when an anti-EGFR therapy is bound to EGFR, which may then lead to resistance to anti-EGFR therapies.^6^

There is conflicting evidence on and, currently, no ongoing prospective trials exploring, the predictive effect of HER2 on resistance to anti-EGFR regimens. For example, several studies reported patients who were HER2-positive receiving anti-EGFR agents had significantly shorter progression-free survival (PFS) (2.5 vs. 6.7 months^14^; 3.1 vs. 5.6 months^15^; 2.8 vs. 8.1 months^16^) and overall survival (OS) (10.2 vs. 17.1 months^17^; 4.2 vs. 13 months^14^) compared with patients who were HER2-negative. In contrast, other studies reported nonsignificant reductions in PFS (5.7 months vs. 7.0 months^11^) and OS (10.1 vs. 13.5 months^15^), or no difference in PFS^18^ or OS.^11,18^

There is a need for a comprehensive review and synthesis of existing literature as early testing for *HER2* amplification in mCRC may identify these patients, potentially sparing them from ineffective treatments. The aim of this systematic review and meta-analysis was to quantitatively assess current evidence on the prognostic or predictive effect of *HER2* amplification/overexpression on anti-EGFR treatment outcomes in patients with *RAS* WT mCRC.

## Materials and Methods

### Systematic Literature Review

A systematic review of MEDLINE, Embase, and Cochrane Library covering 2001 to 2021 was conducted between June and August, 2021 in accordance with PRISMA guidelines.^19^ Primary studies evaluating PFS, overall response rate (ORR), and OS in HER2-positive compared with HER2-negative patients with RAS WT mCRC who received anti-EGFR treatments (cetuximab and panitumumab) and whose HER2 status was determined by immunohistochemistry (IHC), in situ hybridization (ISH), or tissue-based next-generation sequencing (NGS), were included (search criteria are shown in Supplementary Table S1).

Studies enrolling patients with stage IIIA CRC or earlier stages were excluded as were studies of patients with locally advanced and metastatic CRC (stage IIIB and above) treated with interventions other than anti-EGFR treatments with or without chemotherapy, interventions used in adjuvant/neo-adjuvant or other setting, and studies that did not stratify results by HER2 status. Titles and abstracts were reviewed by 2 researchers independently using Covidence platform to identify papers of potential importance. Two researchers then performed an independent full-text review of selected papers based on inclusion/exclusion criteria; where there was discrepancy in selected papers, resolution was facilitated by a 3rd reviewer. Study quality was assessed using the Newcastle-Ottawa scale, which grades studies in terms of population selection, group comparability, and outcomes assessment.^20^

### Meta-Analysis

Hazard ratios (HR) that were directly reported in included studies or calculated HRs (patient-level data that were extracted from Kaplan-Meier [KM] curves) were considered for the meta-analyses. Reconstruction of individual patient data from published KM curves was performed using the Guyot algorithm. HRs were calculated from digitized individual patient data using Cox proportional hazards model. Meta-analyses of proportions (ORR) and HR (PFS, OS) were performed using the random-effects Sidik-Jonkman model to account for the statistical heterogeneity among studies evaluated by I2 statistics.^21,22^ Heterogeneity was defined as follows: no heterogeneity (I2 = 0%-25%), low heterogeneity (I2 = 25%-50%); moderate heterogeneity (I2 = 50%-75%); and high heterogeneity (I2 >75%).

Prespecified sensitivity analyses in this study were: exclusion of 1 study at a time and the generation of influence plots to inform on the statistical heterogeneity of studies; exclusion of outlier studies with moderate/high statistical heterogeneity as assessed using I-square values and heterogeneity plots (ie, Galbraith and L’Abbé plots^23,24^); and exclusion of studies assessing first-line treatment only, to explore the impact of later lines of treatment.

Data analysis was undertaken using Stata 17 (Version 17.0, StataCorp, College Station, Texas 77845, USA). Dichotomous outcomes are presented as odds ratios (OR), and survival outcomes as HR. Summary statistics are presented with 95% CIs.

## Results

### Identification of Studies

**Figure 1.**
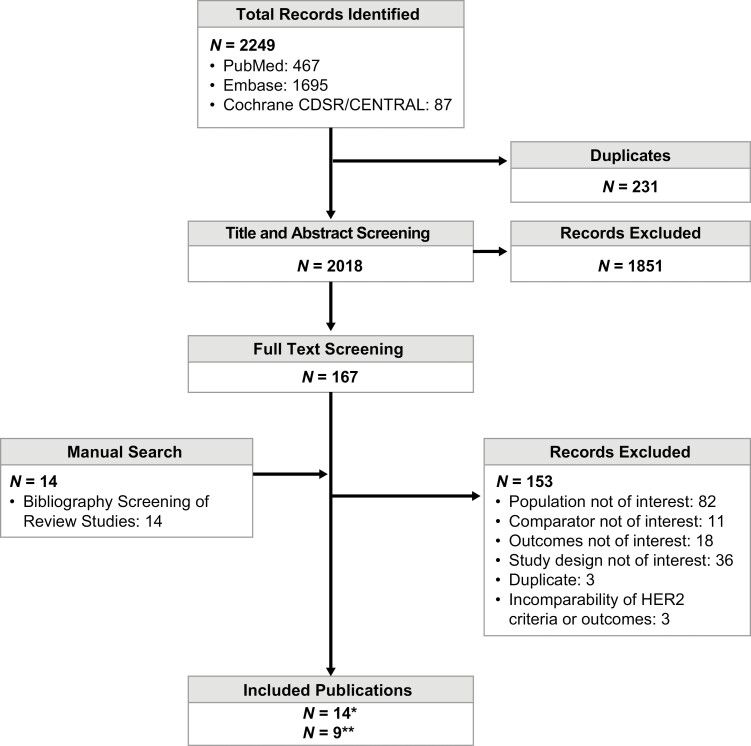
PRISMA flow of citations screened through the SLR process. *Fourteen publications reporting on 12 studies that were subject to meta-analysis feasibility assessment. **Nine publications reporting on 5 studies included in meta-analysis. Abbreviations: Cochrane Library, The Cochrane Central Register of Controlled Trials; CRC, colorectal cancer; Embase, Excerpta Medica Database; HER2, human epidermal growth factor receptor 2; IHC, immunohistochemistry; ISH, in situ hybridization; MEDLINE, Medical Literature Analysis and Retrieval System Online; NGS, next-generation sequencing.

From a total of 2249 references identified across all databases, 167 full-text publications were reviewed (Fig. 1). Of these, 14 publications reporting 12 studies met the inclusion criteria for the systematic literature review and were selected for the feasibility assessment of the OS, PFS, and ORR meta-analysis. Following the feasibility assessment, 9 studies were determined to be unsuitable due to dissimilar HER2-positivity criteria, outcome definitions, or type of outcome measurement, and were excluded. In total, 5 high-quality retrospective cohort studies reported in 9 publications were included in the meta-analyses, representing 594 patients with mCRC (Table 1; Supplementary Table S2 and Table S3).^11,12,15,16,25-29^

**Table 1.** Characteristics of studies included in the meta-analysis.

Study	Sample size	Median age (years)	Male sex (%)	HER2 detection method and positivity criteria	Therapy type	LOT	Median follow-up (months)	Outcome measures
Yagisawa 2021^27^ Sawada 2018^12^	54	65 (HER2+) 64 (HER2-)	20 (HER2+) 61.2 (HER2-)	IHC 3+ or 2+ and FISH HER2/CEP17 ≥2.0	Anti-EGFR alone Anti-EGFR + IRI	1-5L	101.8	PFS^a^, ORR, OS^a^
Jeong 2016/2017^15,25^	142	56	70.4	IHC 3+ or 2+ or IHC scores 3+ or 2+ in ≥50% of cells (HERACLES criteria) and SISH HER2/CEP17 ratio >2.2	CET CET + IRI	2-4L+	13.2	PFS, OS
Sartore-Bianchi 2018/2019^11,26^	216	58.9^b^ (HER2+); 58.4^b^ (HER2-)	75 (HER2+); 68.1 (HER2-)	IHC scores 3+ or 2+ in ≥50% of cells and FISH HER2/CEP17 ≥2.0 in ≥50% of cells (HERACLES criteria)	Anti-EGFR monotherapy+/- CTX	1-5L	50.1 (HER2+); 83.7 (HER2-)	PFS, ORR, OS^a^
Raghav 2016/2019^16,28^	70	57	54.3	ISH positive (HER2/CEP17 ≥2) and NGS ≥4 gene copies identified by an in-house algorithm^c^	CET or PAN CET/PAN + IRI/OX-based CTX	2L/3L	24^d^	PFS
Khelwatty 2021^29^	144	NR	70	IHC score 3+ localized membranous/cytoplasmic HER2 expression ^c^	CET + FOLFOX CET + FOLFIRI	1L	48 (OS 28.5, PFS 19)	PFS, ORR, OS^e^

^a^HR calculated from Kaplan-Meier curves.

^b^Mean age.

^c^Results by NGS detection method and localized membranous HER2 expression were considered for the meta-analyses to be more robust based on consultations with clinical experts.

^d^Assumed from KM curve follow-up.

^e^The HR for OS in Khelwatty et al^29^ was inconsistent, the lower CI was higher than HR value (HR, 0.21 [95% CI, 0.62-0.73]), and it was excluded from the OS analysis. Study authors were contacted to clarify the data but no response was received.

Abbreviations: 1L/2L/3L/4L/5L, first-/ second-/ third-/ fourth-/ fifth-line; CEP, chromosome enumeration probe; CET, cetuximab; CTX, chemotherapy; DISH, dual in situ hybridization; EGFR, epidermal growth factor receptor; FISH, fluorescence in situ hybridization; FOLFIRI, folinic acid, fluorouracil, irinotecan; FOLFOX, folinic acid, fluorouracil, oxaliplatin; HER2+, HER2-positive group; HER2-, HER2-negative group; IHC, immunohistochemistry; IRI, irinotecan; ISH, in situ hybridization; LOT, line of treatment; NGS, next-generation sequencing; NR, not reported; ORR, overall response rate; OS, overall survival; OX, oxaliplatin; PAN, panitumumab; PFS, progression-free survival; SISH, silver in situ hybridization.

### Meta-Analysis Population

Although there was some heterogeneity between studies in terms of patient characteristics (Table 1), no outlier study was identified among the assessed parameters using the box plot method (age, sex, and follow-up period). Median age ranged from 56 years to 64 years, proportion of males from 54.4% to 71.3%, and median duration of follow-up from 13.2 months to 101.8 months across included studies. Some heterogeneity in the HER2 detection method and HER2-positivity criteria utilized in each study was identified. All patients received anti-EGFR treatment, either as monotherapy or in combination with standard chemotherapy, and with the exception of Khelwatty et al,^29^ across different lines of therapy (Table 1).

### Progression-Free Survival

**Figure 2.**
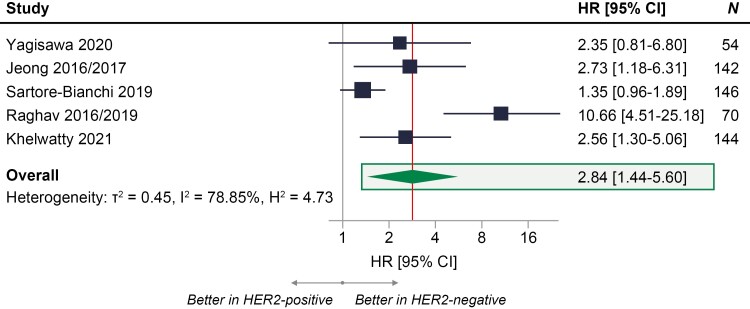
Meta-analysis of PFS with anti-EGFR treatment in patients with RAS WT mCRC who were HER2-positive compared with patients with mCRC who were HER2-negative.^11,12,15,16,25-29^ HR = 1 signifies no statistically significant differences between HER2-positive and HER2-negative groups in the risk of death or progression on anti-EGFR treatment (represented by the gray vertical line); HR > 1 signifies higher risk of death or progression on anti-EGFR treatment in HER2-positive group compared with HER2-negative group; HR < 1 signifies higher risk of death or progression on anti-EGFR treatment in HER2-negative group compared with HER2-positive group; the exact effect size of PFS for the meta-analysis is represented by the vertical red line. Abbreviations: CRC, colorectal cancer; EGFR, epidermal growth factor receptor; HER2, human epidermal growth factor receptor 2; HR, hazard ratio; mCRC, metastatic colorectal cancer; PFS, progression-free survival; WT, wild-type.

Overall, 5 studies reported data on PFS (*n* = 556 patients) using hazard ratios or KM curves and were included in the base-case analysis.^11,12,15,16,25-29^ Results from the meta-analysis of these 5 studies showed there was a 2.84 times higher risk of death or progression (95% CI, 1.44-5.60) in patients with HER2-positive RAS WT mCRC treated with anti-EGFR regimens compared with those who were HER2-negative (Fig. 2).

**Figure 3.**
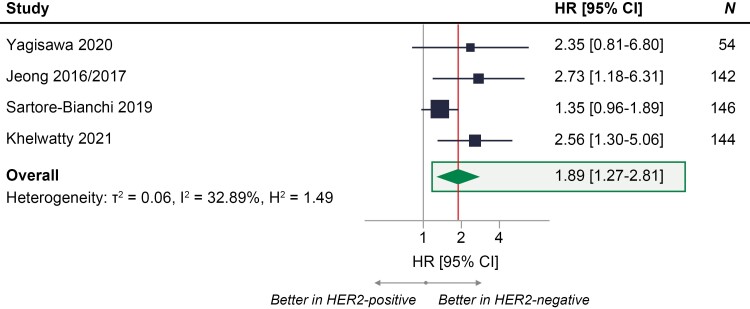
Sensitivity analysis excluding outlier study (Raghav 2016/2019) from meta-analysis of PFS with anti-EGFR treatment in patients with RAS WT mCRC who were HER2-positive compared with patients with mCRC who were HER2-negative.^11,12,15,25-27,29^ HR = 1 signifies no statistically significant differences between HER2-positive and HER2-negative groups in the risk of death or progression on anti-EGFR treatment (represented by the gray vertical line); HR > 1 signifies higher risk of death or progression on anti-EGFR treatment in HER2-positive group compared with HER2-negative group; HR < 1 signifies higher risk of death or progression on anti-EGFR treatment in HER2-negative group compared with HER2-positive group; the exact effect size of PFS for the meta-analysis is represented by the vertical red line. Abbreviations: CRC, colorectal cancer; EGFR, epidermal growth factor receptor; HER2, human epidermal growth factor receptor 2; HR, hazard ratio; mCRC, metastatic colorectal cancer; PFS, progression-free survival; WT, wild-type.

The Galbraith heterogeneity plot indicated Raghav et al^16,28^ as a statistical outlier study due to a high value for HR. Hence, a sensitivity analysis excluding Raghav et al^16,28^ was performed. When Raghav et al^16,28^ was excluded, study heterogeneity reduced from 79% (high heterogeneity) to 33% (low heterogeneity). There was a 1.89 times higher risk of death or progression (95% CI, 1.27-2.81) in patients who were HER2 positive, showing that exclusion of this study did not impact the direction of the base-case results (Fig. 3).

Influence plots, developed by excluding 1 study at a time from the base case, indicated the robustness of the base-case results as there remained a statistically significant higher risk of death or progression in patients with HER2-positive, compared with HER2-negative, RAS WT mCRC treated with anti-EGFR regimens (Supplementary Fig. S1). To explore the impact of later lines of treatment, Khelwatty et al^29^ (which assessed first-line anti-EGFR therapy) was excluded in a prespecified sensitivity analysis. In this sensitivity analysis, there was a 2.97 times higher risk of death or progression (95% CI, 1.25-7.06) in patients who were HER2-positive, confirming the consistency of the study results (Supplementary Fig. S2). Analysis of PFS using different statistical methods (ie, a different random-effects model [DerSimonian-Laird] and a fixed effects model) remained aligned with the base case (HR [95% CI]: 2.86 [1.40-5.82] and 1.99 [1.53-2.59], respectively; Supplementary Fig. S3).

### Overall Response Rate

**Figure 4.**
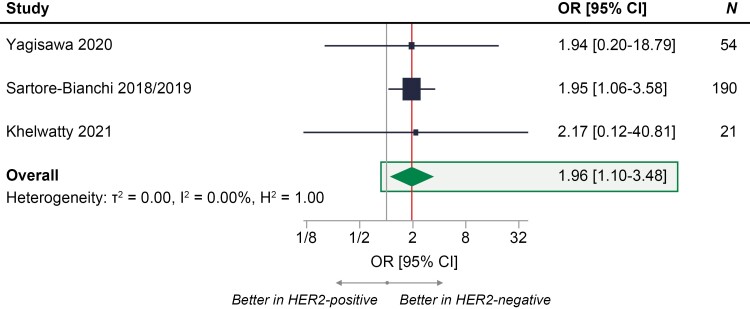
Meta-analysis of ORR to anti-EGFR treatment in patients with RAS WT mCRC who were HER2-positive compared with patients with mCRC who were HER2-negative.^11,12,26,27,29^ OR = 1 signifies no statistically significant differences between HER2-positive and HER2-negative groups in response to anti-EGFR treatment (represented by the gray vertical line); OR < 1 signifies higherodds of response to anti-EGFR treatment in HER2-positive group compared with HER2-negative group; OR > 1 signifies higher odds of response to anti-EGFR treatment in HER2-negative group compared with HER2-positive group; the exact effect size of ORR for the meta-analysis is represented by the vertical red line. Abbreviations: CRC, colorectal cancer; EGFR, epidermal growth factor receptor; HER2, human epidermal growth factor receptor 2; mCRC, metastatic colorectal cancer; OR, odds ratio; ORR, objective response rate; WT, wild-type.

Based on meta-analysis of 3 studies reporting ORR (*n* = 265 patients),^11,26,27,29^ the odds of response to anti-EGFR treatment were almost 2 times higher in patients with mCRC who were HER2-negative compared with HER2-positive (OR [95% CI], 1.96 [1.10-3.48]) (Fig. 4). Influence plots showed that the ORR results remained statistically significant (favoring HER2-negative) when Yagisawa et al^27^ or Khelwatty et al^29^ was excluded; however, the results were not statistically significant when Sartore-Bianchi et al^11,26^ was excluded due to that study having contributed the largest weight (Supplementary Fig. S4).

In a sensitivity analysis exploring the impact of later lines of therapy Khelwatty et al,^29^ the odds of response to anti-EGFR treatment remained almost 2 times higher in patients who were HER2-negative compared with HER2-positive (OR [95% CI], 1.95 [1.08-3.51]; Supplementary Fig. S4). The results of the ORR meta-analysis performed using a fixed-effect model and a different random-effect model (DerSimonian-Laird) remained aligned with the base case (OR [95% CI], 1.96 [1.10-3.48]).

### Overall Survival

**Figure 5.**
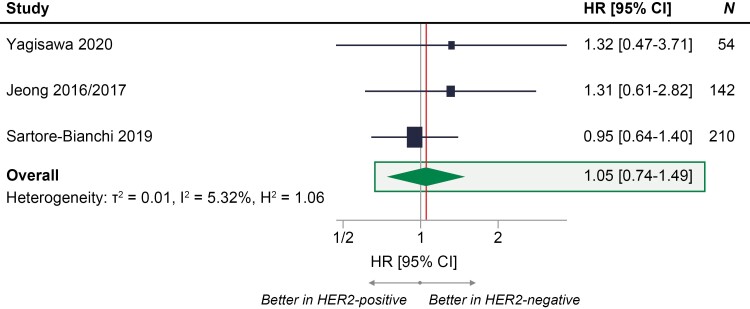
Meta-analysis of OS with anti-EGFR treatment in patients with RAS WT mCRC who were HER2-positive compared with patients with mCRC who were HER2-negative.^11,12,15,25-27^ HR = 1 signifies no statistically significant differences between HER2-positive and HER2-negative groups in response to anti-EGFR treatment (represented by the gray vertical line); HR < 1 signifies higher odds of response to anti-EGFR treatment in HER2-positive group compared with HER2-negative group; HR > 1 signifies higher odds of response to anti-EGFR treatment in HER2-negative group compared with HER2-positive group; the exact effect size of OS for the meta-analysis is represented by the vertical red line. Abbreviations: CRC, colorectal cancer; EGFR, epidermal growth factor receptor; HER2, human epidermal growth factor receptor 2; HR, hazard ratio; mCRC, metastatic colorectal cancer; OS, overall survival; WT, wild-type.

Three studies reported data on OS and were included in the base-case analysis (*n* = 406 patients).^11,15,25-27^ The meta-analysis showed that there was no statistically significant difference in OS between patients with HER2-positive, compared with HER2-negative, RAS WT mCRC (OR [95% CI], 1.05 [0.74-1.49]) (Fig. 5). Sensitivity analyses also showed consistent results, indicating that there was no statistically significant survival benefit with anti-EGFR therapies in patients with RAS WT HER2-positive mCRC.

## Discussion

While well established in other tumor types, such as breast cancer and gastric cancer, HER2 overexpression or amplification has recently emerged as an important and actionable oncogenic driver in CRC that can be screened for and identified with diagnostic tools, and then targeted using biomarker-directed therapy. To the best of our knowledge, this is the first meta-analysis to consolidate and quantify the likely prognostic, possibly predictive effect of HER2 among patients with RAS WT mCRC treated with anti-EGFR therapies. Anti-EGFR therapies were found to be less effective in patients with HER2-positive mCRC than in patients with HER2-negative mCRC, as demonstrated by a statistically significant 2.84-fold higher risk of death or progression, and the almost 2 times higher odds of achieving response to anti-EGFR treatment in HER2-negative patients compared with HER2-positive patients. There was no statistically significant impact of HER2 positivity on OS in patients receiving anti-EGFR therapies. The direction of PFS, ORR, and OS results remained unchanged in most of the sensitivity analyses, confirming the robustness of the base-case estimates.

Our study took a comprehensive and rigorous approach to identify all relevant publications and quantify the impact on clinical outcomes when HER2-positive patients were treated with anti-EGFR regimens. The findings are consistent with a number of previous studies which have suggested that there is an association between HER2 overexpression/amplification and resistance to anti-EGFR therapies in patients with RAS WT mCRC.^11,14,17,30^ Among all identified studies that did not meet the inclusion criteria, or were not able to be included in our meta-analysis due to the definition of HER2 positivity, study population, or outcome estimates reported, there were a few studies worth noting.^14,18^ Consistent with our findings, Martin et al. showed that in patients with mCRC treated with cetuximab or panitumumab, HER2 amplification was associated with poorer outcomes (PFS and OS) whereas no, or slight, HER2 amplification was associated with better outcomes.^14^ Martin et al. was excluded as the study used a variety of definitions for HER2 positivity (eg, copy gain number), which were dissimilar to the studies included in our meta-analysis.^14^ In contrast, Battaglin et al reported that HER2 amplification was not associated with any differences in OS or PFS in patients with mCRC treated first-line with either bevacizumab or cetuximab.^18^ Battaglin et al was excluded as it included a significant proportion of HER2-negative patients with RAS mutations (30.6%)^18^ and RAS mutations are also associated with resistance to anti-EGFR therapies.^8,9^

A notable finding from our analysis was that the HR (comparing HER2 positive with HER2 negative) for PFS reported in each of the included studies varied widely (from 1.35 to 10.66).^11,16,26,28^ Possible explanations for this finding could be differences in types of regimens or lines of therapy used, as greater proportions of patients treated with anti-EGFR as monotherapy, or treated in first-line, may contribute to longer PFS. For example, Raghav et al (which had the greatest HR; 10.66),^16,28^ compared with Sartore-Bianchi et al (which had the smallest HR; 1.35),^11,26^ had smaller proportions of patients receiving anti-EGFR as monotherapy (6.3% vs. 12.6%) and greater proportions of patients receiving anti-EGFR therapies in later lines (all patients on second-/third-line vs. ~50% of patients on first-line therapy). Differences in HER2-testing methodologies (eg, IHC, fluorescence in situ hybridization [FISH], and NGS) may also have resulted in differences in patient populations. In addition, Raghav et al defined HER2-positivity as ≥4 gene copies (identified by NGS and an in-house algorithm),^16,28^ whereas Sartore-Bianchi et al used HERACLES criteria (ie, based on IHC and FISH).^11,26^ As such, the reasons for the large range in HR for PFS among these studies are not clear. Nevertheless, even with some heterogeneity in HR estimates in these studies, sensitivity analyses showed that the likely prognostic, possibly predictive effect of HER2 positivity on anti-EGFR regimens is robust.

In addition to HER2, there are other well-known genetic alterations that are associated with resistance to anti-EGFR therapies, such as mutations in downstream effectors of the EGFR signaling pathway.^17,31^ For example, *BRAF*^*V600E*^ mutations (comprising 8%-12% of patients with mCRC) are predictive of a poorer response to anti-EGFR therapy^32^ as are *KRAS/NRAS* mutations.^8,9^

In our study, we found no impact on OS when patients with HER2-positive disease were treated with anti-EGFR therapies and this is consistent with similar findings for those other genomic biomarkers that confer resistance.^8,9,32,33^ The lack of impact on OS could be due to multiple confounding factors, such as insufficient or variable follow-up time, or subsequent treatment received.

Recently, interest has grown in HER2-targeting strategies for patients with mCRC, where amplification or overexpression of HER2 has been found to occur in approximately 3%-5% of patients.^34-36^ Determination of tumor gene status for *KRAS/NRAS* and *BRAF* mutations and *HER2* amplifications is recommended in NCCN Guidelines.^3,4^ However, the frequency of HER2 testing in clinical practice may have been limited^37^ prior to the recent US Food and Drug Administration approval of tucatinib as the first HER2-targeted agent approved in combination with trastuzumab for patients with HER2-amplified and *RAS*/*BRAF* WT mCRC.^3,4^ Tucatinib with trastuzumab showed tolerability and had a confirmed ORR of 38.1% in the MOUNTAINEER phase II trial in patients with HER2+ *RAS* WT mCRC.^38^ Shortly after US Food and Drug Administration approval, tucatinib was included in NCCN Guidelines as a recommended treatment for patients with HER2-amplified and *RAS/BRAF* WT mCRC.^3,4^ Updated guidelines, together with the findings of this study, further highlight the importance of HER2 testing before treatment initiation to inform treatment decisions. At the same time, large randomized clinical trials with longer follow-up can help further investigate the impact of HER2 positivity on OS and such trials (eg, MOUNTAINEER-03, NCT05253651; and SWOG S1613, NCT03365882) are currently active or recruiting patients.

### Limitations

The following limitations should be considered when interpreting the results of this analysis. First, the studies included in this meta-analysis were of a retrospective cohort design rather than prospective studies; although, they were all considered high-quality by the Newcastle-Ottawa scale. Second, median follow-up time varied across the studies included (from 13 months to 101.8 months); although, HR was used as an outcome measure, which is less sensitive to follow-up duration. Third, regimens used in included studies often comprised combination treatments with standard chemotherapies. Therefore, it was not possible to account for the impact of standard chemotherapies on the pooled effect size given the absence of data stratified per treatment regimen, which can be a source of uncertainty around the meta-analysis estimates. Sartore-Bianchi et al reported that the overall negative impact of HER2 positivity was more pronounced in later lines of therapy, where the confounding impact of chemotherapy was less pronounced.^11^ However, as there were no data on outcomes by the line of therapy in the studies included, it was not possible to assess its impact in this analysis.

## Conclusions

In patients with HER2 overexpressing/amplified RAS WT mCRC, anti-EGFR therapies are associated with worse PFS and ORR; however, there was no impact on OS. While these findings do not fully account for any impact of line of therapy or confounding chemotherapy agents, they support the preclinical evidence that HER2 overexpression/amplification is associated with resistance to anti-EGFR therapies. As such, HER2 testing should be performed before initial treatment consideration, to help inform treatment decisions and optimize outcomes for patients with HER2 amplified mCRC. Further prospective validation of these findings is warranted.

## Supplementary Material

oyad200_suppl_Supplementary_Material

## Data Availability

All data generated or analyzed during this study are included in this published article or in cited literature.
