# Supplementary material for: Impact of Anti-EGFR Therapies on HER2-Positive Metastatic Colorectal Cancer: A Systematic Literature Review and Meta-Analysis of Clinical Outcomes
Source: Oncologist. 2023 Jul 18;28(10):885–93. doi: 10.1093/oncolo/oyad200 (PMC10546818; doi:10.1093/oncolo/oyad200)
Supplement: oyad200_suppl_Supplementary_Material [file oyad200_suppl_supplementary_material.pdf]

## SUPPLEMENTAL MATERIALS

**Supplementary Table S1.** Study search criteria for the systematic literature review.

| PICOS | Inclusion criteria |
|---|---|
| POPULATION | • Adult patients with metastatic RAS WT CRC whose HER2 status was determined by IHC, ISH, or NGS |
| INTERVENTION/EXPOSURE | • HER2+ (positive) patients who received anti-EGFR therapies |
| COMPARATOR/REFERENCE STANDARD | • HER2- (negative) patients who received treatment with anti-EGFR therapies with or without chemotherapy |
| OUTCOMES | • OS, PFS, and ORR |
| STUDY DESIGN | • Prospective/ retrospective interventional studies (cohort, case-control) |

Abbreviations: CRC, colorectal cancer; EGFR, epidermal growth factor receptor; HER2, human epidermal growth factor receptor 2; IHC, immunohistochemistry; ISH, in-situ hybridization; NGS, next-generation sequencing; ORR, overall response rate; OS, overall survival; PFS, progression-free survival; PICOS, Population, Intervention, Comparison and Outcomes; WT, wild-type.

**Supplementary Table S2.** Reported outcomes in studies included in the meta-analysis.

| Study | HER2+ | HER2- |
|---|---|---|
| Yagisawa 2021[27] | | |
| n | 5 | 49 |
| PFS (months), median (95% CI) | 2.2 (0.3-4.1) | 5.1 (4.0-6.1) |
| ORR, % | 20.0 | 32.7 |
| OS (months), median (95% CI) | 34.3 (11.7-56.8) | 38.1 (25.2-50.9) |
| Jeong 2016/2017[15,25] | | |
| n | 7 | 135 |
| PFS (months), median (95% CI) | 3.1 (NR) | 5.6 (NR) |
| PFS HR (95% CI) | 2.73 (1.18-6.31) | |
| ORR, % | NR | NR |
| OS (months), median (95% CI) | 10.1 (NR) | 13.5 (NR); |
| OS HR (95% CI) | 1.31 (0.61-2.82) | |
| Sartore-Bianchi 2018/2019[11,26] | | |
| PFS (months), n, median (95% CI) | 57, 5.7 (4.9–6.0) | 89, 7 (6.0–8.0) |
| PFS HR (95% CI) | 1.35 (0.96-1.89) | |
| ORR, n, % | 77, 31.2 | 113, 46.9 |
| OS (months), n, median (95% CI) | 94, 44.6 (35.2-49.4) | 116, 43.7 (34.3-49.7) |
| Raghav 2016/2019[16,28] | | |
| PFS (months), n, median (95% CI) | 16, 2.8 (1.7-4.0) | 54, 9.3 (8.7-9.9) |
| PFS HR (95% CI) | 10.66 (4.5-25.2) | |
| ORR, % | NR | NR |
| OS (months), n, median (95% CI) | 12, Not reached | 46, 60.2 (NR) |
| OS HR (95% CI) | 1.27 (0.4-3.7) | |

| | | | |
|---|---|---|---|
| Khelwatty 2021[29] | | | |
| n | 39 | | 105 |
| PFS (months), median (95% CI) | NR | | NR |
| PFS HR (95% CI) | | 2.56 (1.30–5.06) | |
| ORR, % | NR | | NR |
| OS (months), median (95% CI) | NR | | NR |
| OS HR (95% CI) | | 0.21 (0.62-0.73) | |

Yagisawa 2021 reported two different median PFS values (one reported in the body of manuscript and within Figure 4, and a second reported only in the Title of Figure 4). In this study, we utilized the former (shown in the table above) as they were consistent in two separate places in Yagisawa 2021. The discrepancy in reported median PFS values does not impact the results of this meta-analysis as hazard ratios were used, not median PFS.

HER2+, HER2-positive group; HER2-, HER2-negative group; NR, not reported; ORR, overall response rate; OS, overall survival; PFS, progression-free survival.

**Supplementary Table S3.** Characteristics of studies identified for the meta-analysis.

| Study | Sample size | Geographic scope | Other mutations reported | Tumor sidedness | |
|---|---|---|---|---|---|
| | | | | Left-sided | Right-sided |
| Yagisawa 2021[27] | 370 | Japan (single-center) | RAS/BRAF WT: HER2+ 73%, HER2- 41%<br>RAS mutant: HER2+ 27%, HER2- 37%<br>BRAF V600E mutant: HER2+ 0%, HER2- 4% | HER2+ 73%, HER2- 78% | HER2+ 27%, HER2- 22% |
| Sawada 2018[12] | 142 | Japan (single-center) | RAS/BRAF WT: 100% | HER2+ 60%, HER2- 36.7% | HER2+ 20%, HER2- 12.2% |
| Jeong 2016/2017[15,25] | 142 | South Korea (single-center) | RAS/BRAF WT: 100% | 77.8% | 22.2% |
| Sartore-Bianchi 2018/2019[11,26] | 216 | Italy (multicenter) | KRAS exon 2 WT | HER2+ 89.5%, HER2- 80.8% | HER2+ 10.5%, HER2- 19.1% |
| Raghav 2016/2019[16,28] | 70 | USA (multicenter) | TP53-mutant: 47.6%<br>APC-mutant: 23.1% | HER2+ 93.8%, HER2- 85.2% | HER2+ 6.2%, HER2- 14.8% |
| Khelwatty 2021[29] | 144 | UK (multicenter) | RAS WT | NR | NR |

Yagisawa 2021 reported patient characteristics for the entire cohort, i.e., without stratification for patients treated with anti-EGFR.

Khelwatty 2021 reported co-expressions of HER3 and HER4 with different proportion of patients affected depending on expression criteria (% positive tumor cells, and intensity).

Abbreviations: HER2+, HER2-positive group; HER2-, HER2-negative group; NR, not reported; WT, wild-type.

**Supplementary Fig. S1.** Influence plots developed by excluding 1 study at a time from the base-case analysis of PFS data.

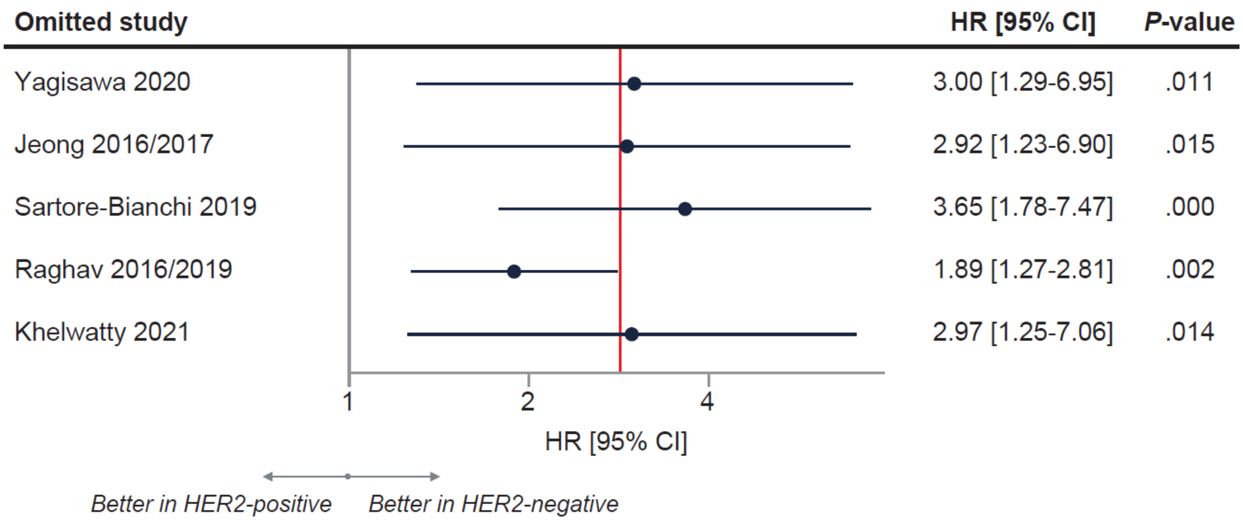


HR = 1 signifies no statistically significant differences between HER2-positive and HER2-negative groups in the risk of death or progression on anti-EGFR treatment (represented by the gray vertical line); HR > 1 signifies higher risk of death or progression on anti-EGFR treatment in HER2-positive group compared with HER2-negative group; HR < 1 signifies higher risk of death or progression on anti-EGFR treatment in HER2-negative group compared with HER2-positive group; The exact effect size of PFS for the meta-analysis is represented by the vertical red line.

Abbreviations: CI, confidence interval; EGFR, epidermal growth factor receptor; HER2, human epidermal growth factor receptor 2; HR, hazard ratio; PFS, progression-free survival.

**Supplementary Fig. S2.** Sensitivity analysis excluding outlier study (Khelwatty, 2021) from meta-analysis of PFS with anti-EGFR treatment in patients with RAS WT mCRC who were HER2-positive compared with patients with mCRC who were HER2-negative.

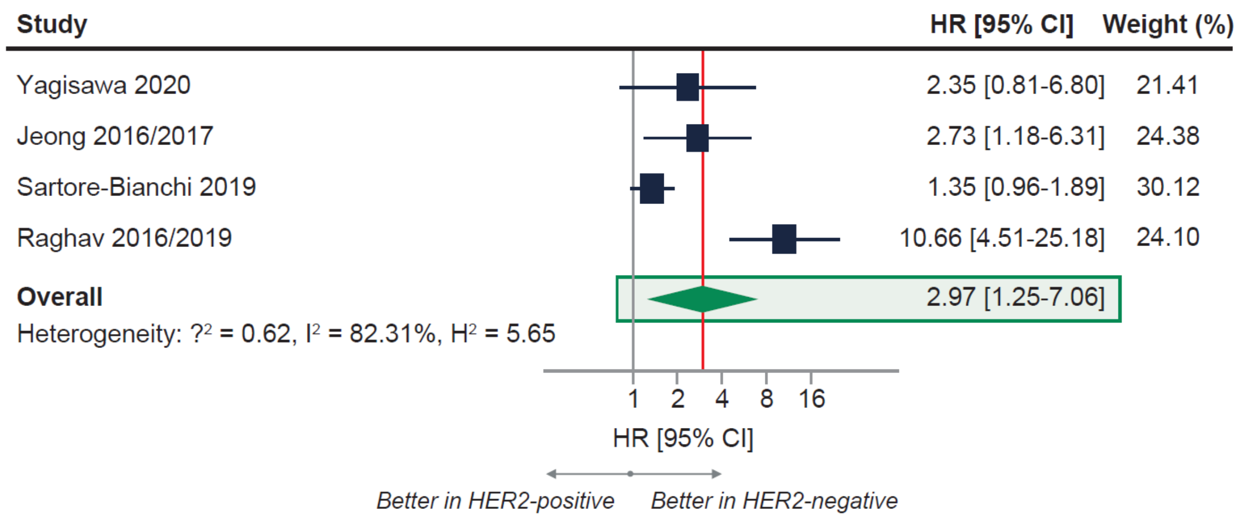


HR = 1 signifies no statistically significant differences between HER2-positive and HER2-negative groups in the risk of death or progression on anti-EGFR treatment (represented by the gray vertical line); HR > 1 signifies higher risk of death or progression on anti-EGFR treatment in HER2-positive group compared with HER2-negative group; HR < 1 signifies higher risk of death or progression on anti-EGFR treatment in HER2-negative group compared with HER2-positive group; the exact effect size of PFS for the meta-analysis is represented by the vertical red line.

Abbreviations: CI, confidence interval; EGFR, epidermal growth factor receptor; HER2, human epidermal growth factor receptor 2; HR, hazard ratio; mCRC, metastatic colorectal cancer; PFS, progression-free survival; WT, wild-type.

**Supplementary Fig. S3.** Meta-analysis of PFS with anti-EGFR treatment in patients with RAS WT mCRC who were HER2-positive compared with patients with mCRC who were HER2-negative, (**A**) fixed effects inverse-variance model and (**B**) random-effects DerSimonian-Laird model.

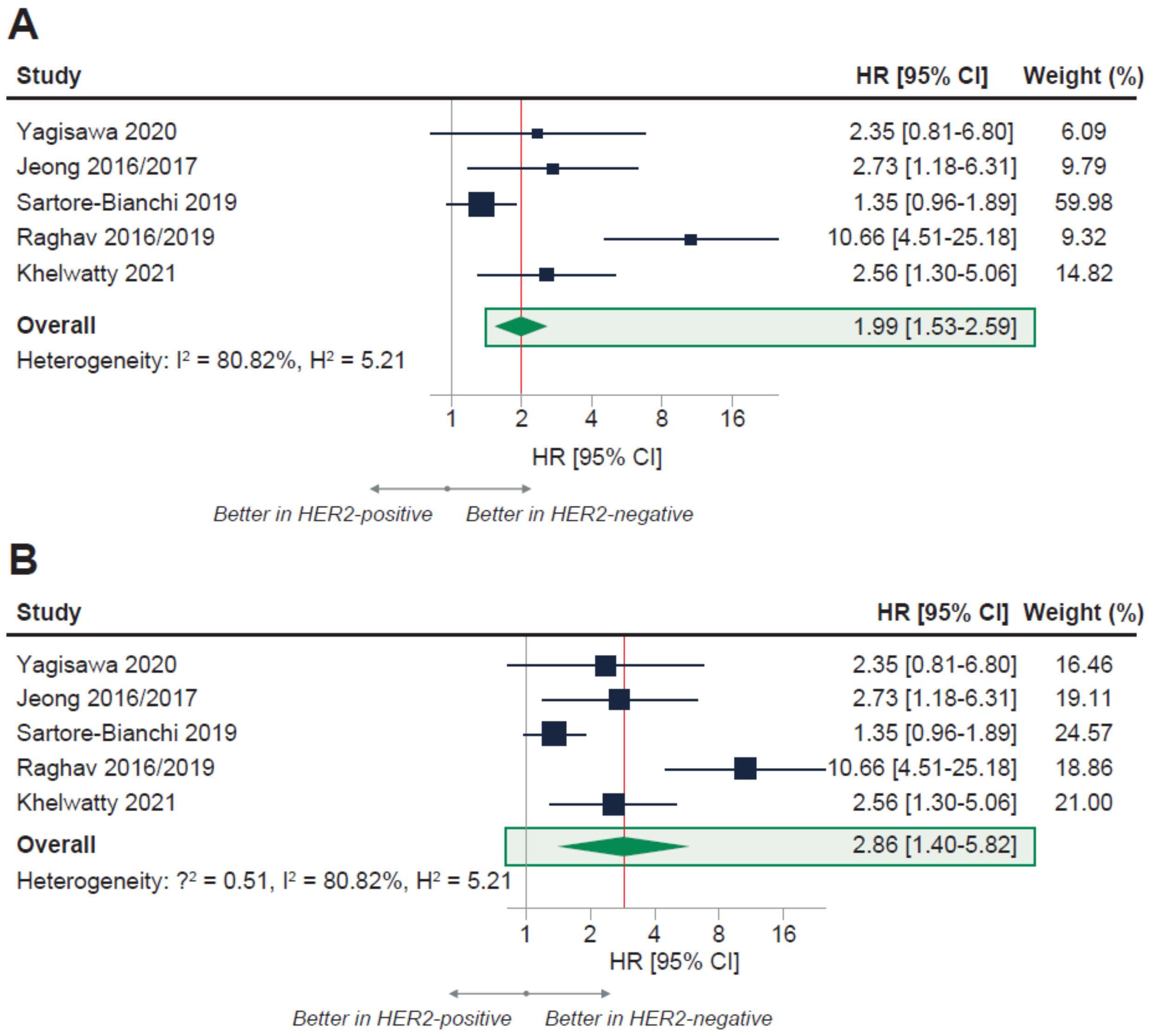


HR = 1 signifies no statistically significant differences between HER2-positive and HER2-negative groups in the risk of death or progression on anti-EGFR treatment (represented by the gray vertical line); HR > 1 signifies higher risk of death or progression on anti-EGFR treatment in HER2-positive group compared with HER2-negative group; HR < 1 signifies higher risk of death or progression on anti-EGFR treatment in HER2-negative group compared with HER2-positive group; the exact effect size of PFS for the meta-analysis is represented by the vertical red line.

Abbreviations: CI, confidence interval; EGFR, epidermal growth factor receptor; HER2, human epidermal growth factor receptor 2; HR, hazard ratio; mCRC, metastatic colorectal cancer; PFS, progression-free survival; WT, wild-type.

**Supplementary Fig. S4.** Influence plots developed by excluding 1 study at a time from the base-case analysis of ORR data.

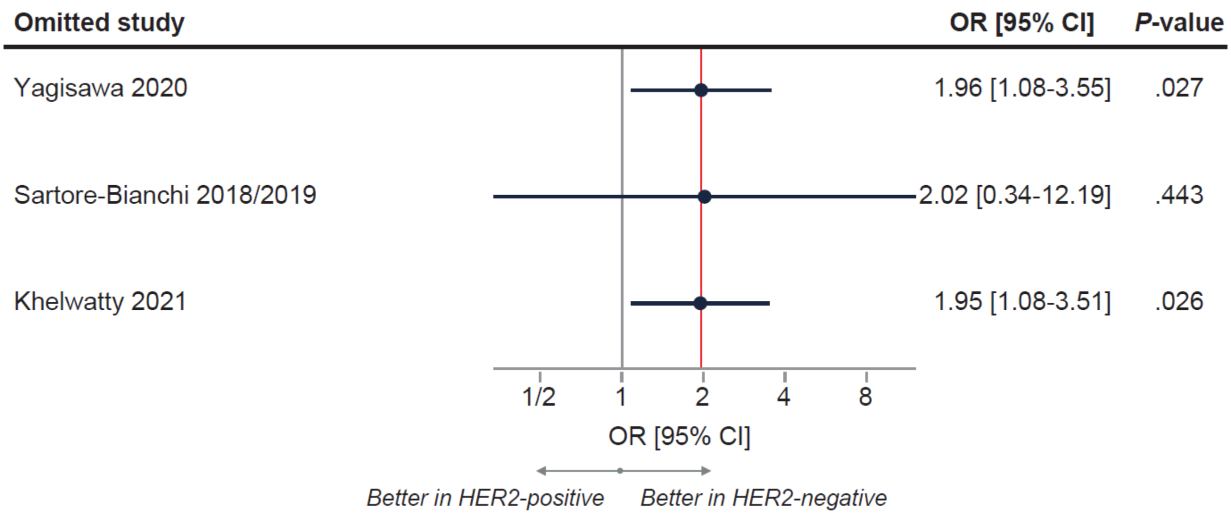


OR = 1 signifies no statistically significant differences between HER2-positive and HER2-negative groups in the risk of death or progression on anti-EGFR treatment (represented by the gray vertical line); OR > 1 signifies higher risk of death or progression on anti-EGFR treatment in HER2-positive group compared with HER2-negative group; OR < 1 signifies higher risk of death or progression on anti-EGFR treatment in HER2-negative group compared with HER2-positive group; the exact effect size of ORR for the meta-analysis is represented by the vertical red line.

Abbreviations: CI, confidence interval; EGFR, epidermal growth factor receptor; HER2, human epidermal growth factor receptor 2; OR, odds ratio; ORR, overall response rate.
